# The Central Role of Oxo Clusters in Zirconium‐Based Esterification Catalysis

**DOI:** 10.1002/smsc.202400369

**Published:** 2024-09-23

**Authors:** Jikson Pulparayil Mathew, Carlotta Seno, Mohit Jaiswal, Charlotte Simms, Nico Reichholf, Dietger Van den Eynden, Tatjana N. Parac‐Vogt, Jonathan De Roo

**Affiliations:** ^1^ Department of Chemistry University of Basel Mattenstrasse 22 Basel 4058 Switzerland; ^2^ Department of Chemical Sciences Indian Institute of Science Education and Research (IISER)‐Mohali Mohali SAS Nagar Punjab 140306 India; ^3^ Department of Chemistry KU Leuven Celestijnenlaan 200F 3001 Leuven Belgium

**Keywords:** esterification catalysis, group 4, MOFs, nanocrystals, pair distribution function analysis, oxo‐cluster, zirconium

## Abstract

Oxo clusters are a unique link between oxide nanocrystals and Metal‐Organic Frameworks (MOFs), representing the limit of downscaling each of the respective crystals. Herein, the superior catalytic activity of ${\text{Zr}}_{12}O_{8}{\left(\text{OH}\right)}_{8}{\left(\text{OOCR}\right)}_{24}$ clusters, compared to zirconium MOF UiO‐66 and ${\text{ZrO}}_{2}$ nanocrystals is shown. Focus is on esterification reactions given their general importance in consumer products and the challenge of converting large substrates. Oxo clusters have a higher surface‐to‐volume ratio than nanocrystals, rendering them more active. For large substrates, for example, oleic acid, MOF UiO‐66 has negligible catalytic activity while clusters provide almost quantitative conversion, a fact we ascribe to limited diffusion of large substrates through the MOF pores. Clusters do not suffer from limited mass transfer and we also obtain high conversion in solvent‐free reactions with sterically hindered alcohols (hexanol, 2‐ethyl hexanol, benzyl alcohol, and neopentyl alcohol). The cluster catalyst can be recovered and shows identical activity when reused. The structural integrity of the cluster is confirmed using X‐ray total scattering and pair distribution function analysis. Moreover, when homogeneous zirconium alkoxides are used as catalysts, the same oxo cluster is retrieved, showing that oxo clusters are the active catalytic species, even in previously assumed homogeneously catalyzed reactions.

## Graphical abstract

This work focuses on exploring the higher catalytic activity of zirconium oxo‐clusters over UiO‐66 and ZrO_2_. The recovered clusters are studied using pair distribution function, nuclear magnetic resonance, and fourier‐transform infrared to get insight into the structural integrity. The retrieval of oxo‐clusters when using zirconium alkoxides as catalyst proves that the active catalytic species is an oxo‐cluster.

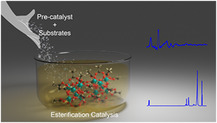

## 1 Introduction

Homogenous and heterogeneous catalyst materials are, in principle, two distinct and well‐defined groups, being either the same phase as the substrate or different. Homogeneous catalysts are metal salts or metal complexes that dissolve in the reaction medium and are most often not recovered. Heterogeneous catalysts are often insoluble powders, dispersed in the reaction medium through stirring, and the powder is easily recovered by filtration or centrifugation. Typical examples of heterogeneous catalysts are 1) bulk oxides or 2) Metal‐Organic Frameworks (MOFs). With the advent of nanocrystal synthesis,^[^ ^1^ ^]^ an opportunity was created to make heterogeneous catalysts more active by increasing their surface area or exposing certain facets.^[^ ^2^, ^3^, ^4^ ^]^ While aggregated or supported nanocrystals still appear as a powder, colloidal nanocrystals (of only a few nanometers in size) form stable dispersions without any light scattering. One can debate whether colloidal nanocrystals belong to the realm of homogeneous or heterogeneous catalysis. They feature a solid–liquid interface but are homogeneously dispersed in solution. To further muddle the definitions, there are claims that certain homogeneous catalysts form colloidal nanocrystals during catalysis.^[^ ^5^ ^]^

**Figure 1:**
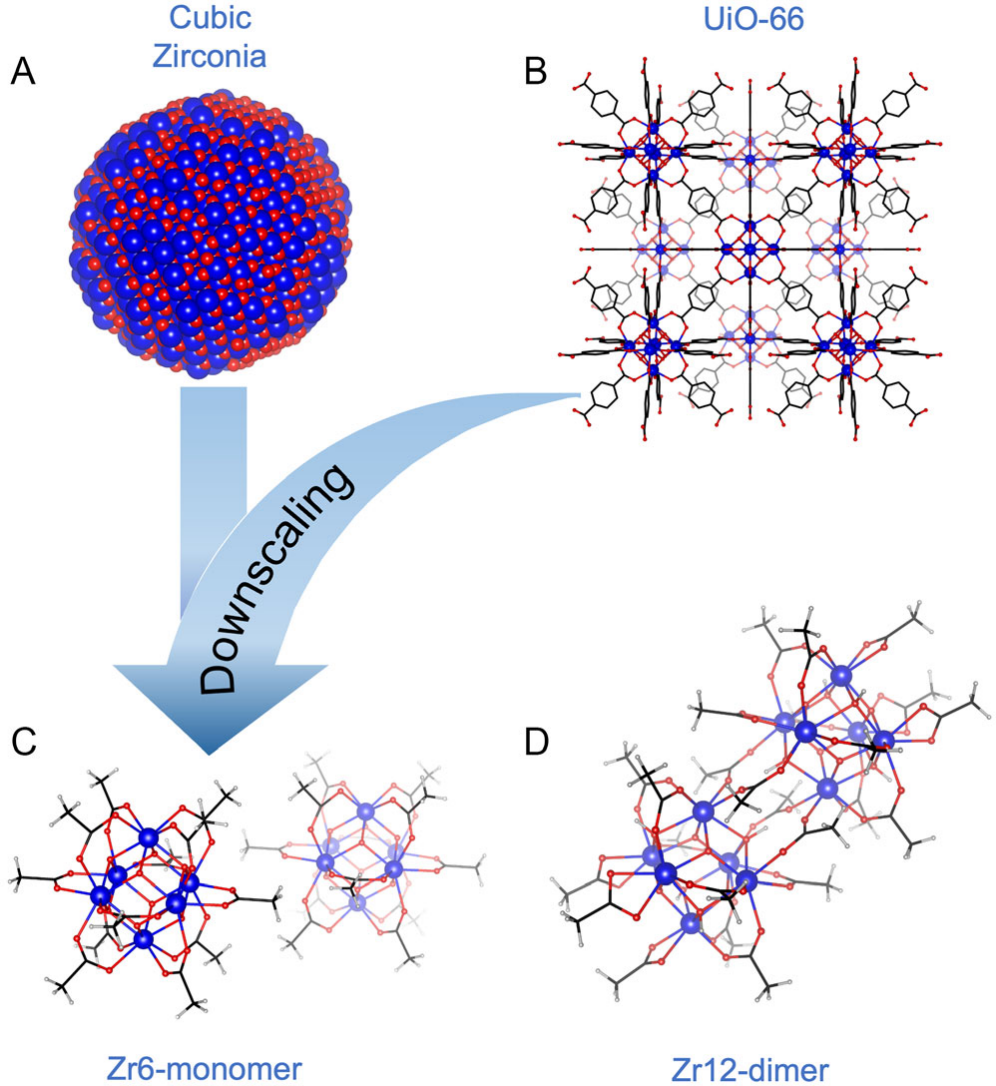
Structural representation of catalysts used in this article. A) Nanocrystal (${\text{ZrO}}_{2}$), B) Metal–organic framework (UiO‐66),^[^ ^84^ ^]^ C) **Zr6** oxo cluster (**Zr6**‐acetate),^[^ ^85^ ^]^ and D) **Zr12** oxo cluster (**Zr12**‐acetate).

When further increasing the surface‐to‐volume ratio of nanocrystals by decreasing their size, one reaches a limit, beyond which, the material would lose its particle character and become a metal complex. For example, in the case of zirconia, we can conceive a spheroidal particle containing six zirconium atoms where the atomic arrangement of zirconium and oxygen is still identical to the one in the cubic crystal structure of zirconia. The surface is capped with (carboxylate) ligands, just like for colloidal nanocrystals. Such objects are known as discrete oxo clusters.^[^ ^6^, ^7^ ^]^ Exactly the same structure is obtained when scaling down zirconium MOF (nano)crystals until only a single secondary building unit is left with monofunctional ligands instead of bifunctional linkers. Discrete oxo clusters thus provide the missing link between oxide nanocrystals and MOFs and their relationship is illustrated in **Figure** **1**. This link is the object of our study.

So far, nanocrystals and MOFs have been extensively researched in the field of catalysis. Nanocrystals are currently exploited in thermal catalysis,^[^ ^8^, ^9^, ^10^ ^]^ electrochemical reactions^[^ ^11^, ^12^, ^13^ ^]^ and photochemical processes.^[^ ^14^, ^15^, ^16^ ^]^ MOFs are developed as heterogeneous catalysts.^[^ ^17^, ^18^, ^19^, ^20^, ^21^, ^22^, ^23^, ^24^, ^25^ ^]^ Their high porosity and high surface area made MOFs highly appealing, either as support or as an intrinsic catalyst.^[^ ^26^ ^]^ In the latter case, the metal atoms in the nodes are the catalytic centers. The steric and electronic properties of the linkers can be engineered to influence the activity of the metal node.^[^ ^27^, ^28^, ^29^ ^]^ Within the group 4 MOFs, the nodes are metal oxo clusters. Group 4 MOFs have been extensively researched due to their high stability, Lewis acidity, and tunable structure.^[^ ^30^, ^31^ ^]^ To improve their catalytic activity, defects are often introduced.^[^ ^32^ ^]^ These MOFs have been employed in hydrolysis reactions,^[^ ^33^, ^34^ ^]^ esterifications,^[^ ^35^, ^36^ ^]^ amide bond formation,^[^ ^37^ ^]^ cyclization reactions,^[^ ^38^ ^]^ asymmetric deacetalization–acetalization reactions,^[^ ^39^ ^]^ the enantioselective Friedel–Crafts Reaction,^[^ ^39^ ^]^ and condensation reactions.^[^ ^40^, ^41^ ^]^ However, the catalytic activity of MOFs seems to be poor toward large and bulky substrates, for which diffusion through the small pores becomes very slow.^[^ ^42^, ^43^, ^44^ ^]^ Research has therefore focused on designing MOFs with larger pore sizes or with smaller crystallite sizes to increase the active surface area and minimize the effect of internal diffusion on reaction kinetics.^[^ ^44^, ^45^, ^46^, ^47^, ^48^ ^]^

To maximize the number of active sites, we and others recently started exploring soluble zirconium and hafnium oxo clusters as homogeneous catalysts. These clusters have an $M_{6}O_{8}$ core, capped with protons and carboxylates. The clusters typically appear either as monomers (${\text{Zr}}_{6}O_{4}{\left(\text{OH}\right)}_{4}{\left(\text{OOCR}\right)}_{12}$), or as dimers (${\text{Zr}}_{12}O_{8}{\left(\text{OH}\right)}_{8}{\left(\text{OOCR}\right)}_{24}$), depending on the steric hindrance of the carboxylate ligand.^[^ ^6^, ^49^ ^]^ Even though polyoxometalate anions have been widely reported in the field of catalysis,^[^ ^50^, ^51^, ^52^ ^]^ neutral oxo clusters are less explored. Zirconium oxo clusters were used for amide bond formation,^[^ ^7^, ^53^ ^]^ amine oxidation,^[^ ^54^ ^]^ hydrogen peroxide activation,^[^ ^55^ ^]^ and proteolysis.^[^ ^56^ ^]^ In addition, oxo clusters are atomically precise objects (in contrast to MOFs or nanocrystals) and thus open exciting opportunities for mechanistic studies.

Here, we propose zirconium and hafnium oxo clusters as highly active catalysts for the esterification of bulky acid or alcohol substrates. Their esters are relevant as biofuels, emollients, plasticizers, and lubricants.^[^ ^57^, ^58^, ^59^, ^60^, ^61^ ^]^ Oxo clusters are by far superior catalysts compared to oxide nanocrystals and MOFs due to their maximal surface‐to‐volume ratio and the absence of diffusion limitations. We further showed that the oxo cluster structure is stable during catalysis, aided by X‐ray total scattering and pair distribution function (PDF) analysis. Even more, we find that oxo clusters are formed when metal alkoxides are employed as pre‐catalysts, thus elucidating the active species in previously reported catalytic reactions.

## 2 Results and Discussion

### 2.1 Superior Catalytic Activity of Oxo Clusters

**Figure 2:**
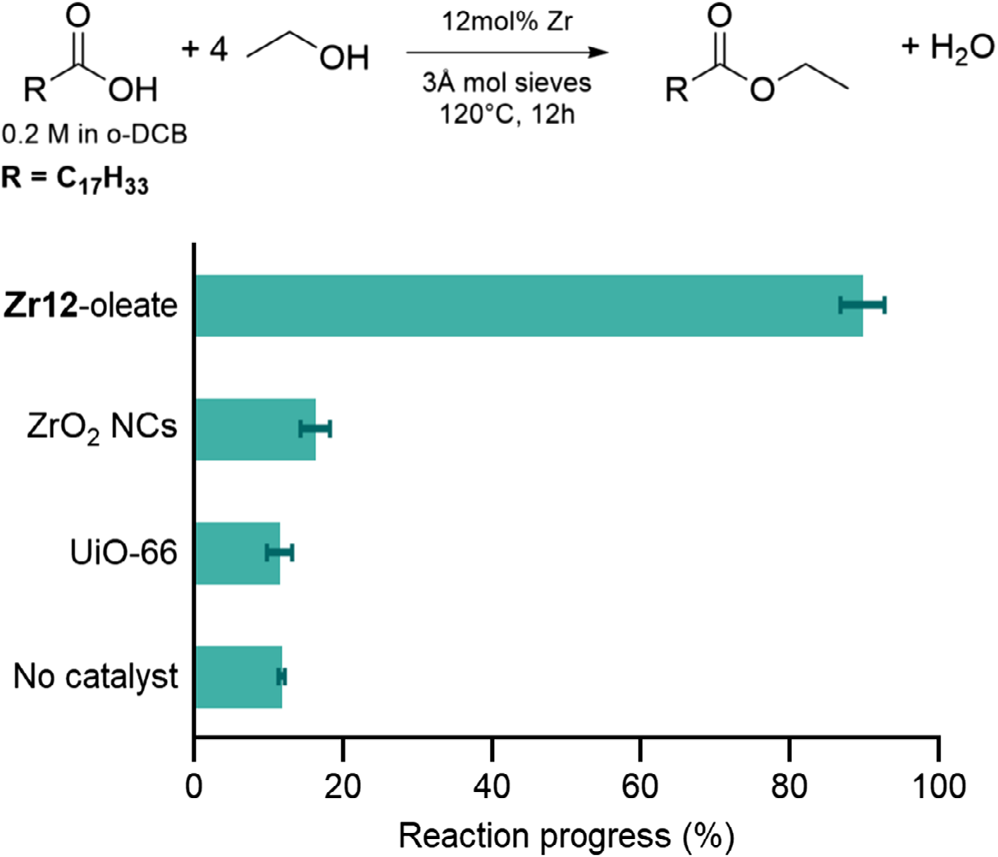
Catalytic esterification of oleic acid with ethanol in *ortho*‐dichlorobenzene (*o*‐DCB). The catalyst is either **Zr12**‐oleate, ${\text{ZrO}}_{2}$ nanocrystals, or the MOF UiO‐66. The reactions were performed in triplicate.

To demonstrate the superior catalytic activity of zirconium oxo clusters with respect to zirconium MOFs and ${\text{ZrO}}_{2}$ nanocrystals, we choose esterification as a model reaction due to its wide application in the pharmaceutical^[^ ^62^ ^]^ and cosmetic industry.^[^ ^63^ ^]^ Esterification is also used for the production of emulsifiers,^[^ ^64^, ^65^ ^]^ plasticizers,^[^ ^66^ ^]^ and biodiesel.^[^ ^67^, ^68^, ^69^, ^70^ ^]^ UiO‐66, ${\text{ZrO}}_{2}$ nanocrystals, and **Zr12** oxo clusters were synthesized according to previously published procedures.^[^ ^49^, ^71^, ^72^, ^73^ ^]^ Oleate was used as the ligand for the oxo clusters and nanocrystals since oleic acid is the first substrate for our chosen catalytic reaction, thus avoiding competition between ligand and catalytic substrate for the surface binding sites, and avoiding the production of unwanted side‐products.^[^ ^74^ ^]^ The oleate‐capped ${\text{ZrO}}_{2}$ nanocrystals, synthesized from zirconium isopropoxide and benzyl alcohol, have an average diameter of 5.6 nm, see Figure S1, Supporting Information. The ^1^H NMR spectrum shows only broadened resonances of bound oleate ligands (Figure S2, Supporting Information).^[^ ^75^, ^76^ ^]^ UiO‐66 was synthesized from ${\text{ZrCl}}_{4}$ and benzene‐1,4‐dicarboxylic acid and dried in air at 70 °C for 4 h and then activated at 110 °C for 20 h to remove residual solvent from the pores. The synthesized MOF was analyzed via pXRD (powder X‐ray diffraction) (Figure S7, Supporting Information) to confirm the crystallinity of the material and was also digested in NaOH and analyzed by ^1^H NMR (Figure S8, Supporting Information) to determine the linker connectivity of UiO‐66. The inorganic fraction was determined using TGA (Thermogravimetric Analysis) (Figure S9, Supporting Information) and together with ^1^H NMR revealed the presence of ≈1.5 missing linkers per formula unit. Quantifying the number of open metal sites is important for catalysis as the substrate (oleate) must bind to the Zr for the reaction to proceed. This stands in contrast to nanocrystals and oxo clusters where oleate was directly bound to Zr as the stabilizing ligand. The oleate‐capped oxo cluster (**Zr12**‐oleate), synthesized from zirconium propoxide and oleic acid, is a dimer of two **Zr6** octahedral clusters (PDF in Figure S3, Supporting Information and ^1^H NMR in Figure S6, Supporting Information). The activity of all three catalysts was evaluated in the esterification of oleic acid with ethanol as a model reaction (**Figure** **2**). 1 mol% of **Zr12** cluster was added to the reaction mixture, amounting to 12 mol% zirconium. Equal amounts of zirconium (12 mol%) were present in the nanocrystal and MOF catalysts. After 12 h of reaction, almost full conversion was obtained when using the cluster as the catalyst, while the ${\text{ZrO}}_{2}$ nanocrystals only featured a slightly higher conversion compared to the reaction without a catalyst. Interestingly, UiO‐66 does not show any appreciable catalytic activity.

The striking difference between nanocrystals and clusters can be partly ascribed to a difference in surface area. In the **Zr12**‐oleate cluster, all zirconium atoms are available at the surface, thus representing a maximal surface to volume ratio of 12 nm^−1^ (calculated by approximating it to a sphere with a radius of 0.25 nm). In 5.6 nm nanocrystals, about 80% of the zirconium atoms are buried beneath the surface.^[^ ^49^ ^]^ If the difference in activity was only due to a different number of catalytic sites, the turnover number (TON, calculation in the SI) for nanocrystals and clusters would be the same, when calculated per surface metal site. However, after subtracting the control (esterification done without adding any catalyst, keeping rest of the conditions the same) from the reaction progress, the TON was 1.9 and 6.5 for nanocrystals and clusters, respectively (see the SI for the calculations). This means that the clusters are also intrinsically more catalytically active. The steric factor could play a role since clusters have a higher surface curvature than nanocrystals and thus ligands are more densely packed on nanocrystals, hampering the approach of the alcohol. The curvature of a sphere is given by the reciprocal of radius; we calculate a curvature of 4 and 0.36 nm^−1^ for cluster and nanocrystal respectively. In addition, clusters can act as both hydrogen bond donors and acceptors since their surface has $\mu_{3}$ oxygen atoms with and without attached hydrogen atoms.^[^ ^6^ ^]^ This makes them ideally suited to activate the alcohol.

**Figure 3:**
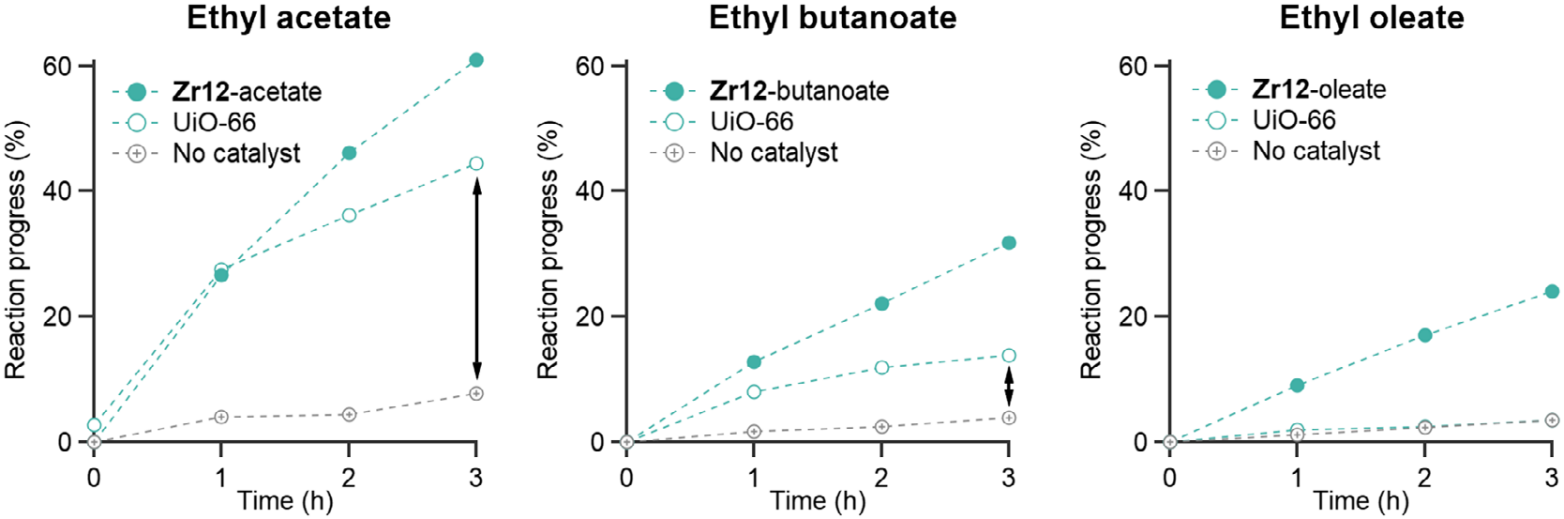
Catalytic esterification, comparing **Zr12** oxo clusters and UiO‐66, for different carboxylic acid substrates. The conditions are identical to Figure 1: 120 °C, 12 mol% zirconium, 0.2 m carboxylic acid, molecular sieves, and four equivalents of ethanol.

The difference in the catalytic activity between MOFs and clusters can be ascribed to node accessibility. In MOFs, diffusion of the large substrate through the pores is slow and catalysis likely only happens at the surface of the MOF microcrystals.^[^ ^77^ ^]^ To verify this hypothesis, we explored the substrate scope; using acetic acid, butanoic acid, and oleic acid as substrates. As cluster catalysts, we used **Zr12**‐acetate, **Zr12**‐butanoate, and **Zr12**‐oleate, respectively, to avoid competition between ligand and the incoming substrate.^[^ ^74^ ^]^ The reaction was monitored for 3 h by taking aliquots at a time interval of 1 h, see **Figure** **3**. For carboxylic acids with longer chain lengths, the reaction progress decreased for all catalysts. However, the reactivity decreased more drastically for the MOF catalyst. While the ethyl acetate yield is considerably higher than the control (without catalyst), this is less so for ethyl butanoate. For ethyl oleate, the conversion using MOFs is equal to the reaction without a catalyst. Therefore, we conclude that the UiO‐66 MOF is a suitable esterification catalyst for small substrates but clusters are superior catalysts for bulky substrates.

To get a deeper insight into the catalytic activity of oxo clusters, we explored the influence of various parameters, see **Table** **1**. Entry (1) represents the 12 h reaction as in Figure 1 while the other entries are recorded after 3 h of reaction. As expected, with increasing equivalents of ethanol, higher yields are obtained, see Entries (2)–(5) (and Figure S10, Supporting Information). The addition of 3 Å molecular sieves also had a positive effect (Entries (3) and (6) (and Figure S11, Supporting Information)), since they could help in the removal of water, formed as a side product during the esterification, pushing the equilibrium to the right. Replacing *ortho*‐dichlorobenzene (*o*‐DCB) with mesitylene does not affect the yield (Entries (3) and (7)).

**Table 1:** The esterification of oleic acid with ethanol, using **Zr12**‐oleate as the catalyst.

Entrya)	Alcohol equiv	Solvent	Sieves	Temp. [°C]	Time [h]	Yield [%]
(1)	4	*o*‐DCB	Yes	120	12	89.6
(2)	2	*o*‐DCB	Yes	120	3	17
(3)	4	*o*‐DCB	Yes	120	3	27
(4)	6	*o*‐DCB	Yes	120	3	37
(5)	10	*o*‐DCB	Yes	120	3	45
(6)	4	*o*‐DCB	No	120	3	18
(7)	4	Mesitylene	Yes	120	3	28

a) The catalyst amount is always 1 mol% dimer with respect to the substrate (oleic acid + oleate), which is equivalent to 12 mol% Zr.

### 2.2 Solventless Esterification Using Higher Alcohols

**Figure 4:**
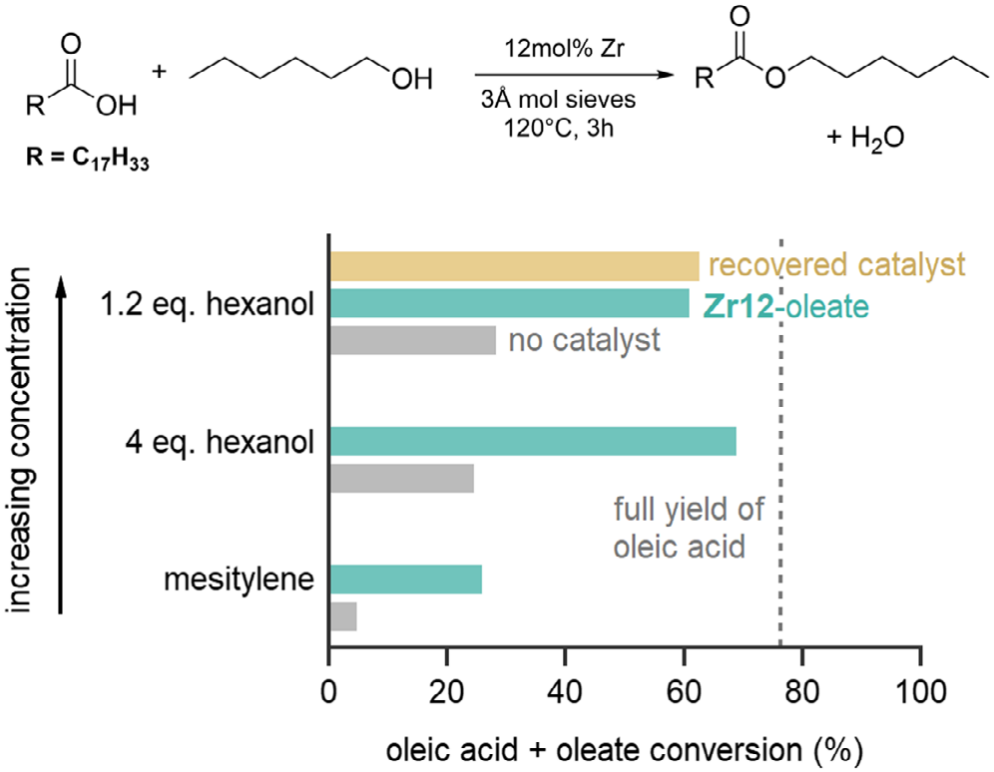
Catalytic esterification of oleic acid with hexanol. The reaction is either done in mesitylene (using four equivalents hexanol), without mesitylene (using four equivalents hexanol), or without mesitylene and a reduced 1.2 equivalents of hexanol. In the latter case, we recovered the catalyst and used this for a second catalytic reaction. The dotted line corresponds to the maximum yield that can be obtained when excluding the oleate ligands on the catalyst surface.

Since reactions without an additional solvent are highly desirable and more sustainable, we explored solvent‐less reactions with high‐boiling alcohols. The latter are also generally more challenging and less reactive than ethanol or methanol. In the case of hexanol, we compared the standard reaction (using four equivalents of hexanol) in mesitylene with a reaction where the solvent was omitted, and another reaction where the amount of hexanol was reduced to 1.2 equivalents. Given that the total volume of the latter reaction mixture decreases, the concentration of carboxylic acid and catalyst increases. While a moderate conversion was observed after only 3 h in mesitylene, 69% yield was obtained in the solvent‐free reaction (**Figure** **4**). The catalyzed yield decreased to 61% for only 1.2 equivalents of hexanol while the yield without catalyst, in this case, increased slightly to 28%. The reaction with 1.2 equivalents of hexanol does have the highest atom economy and is for that reason interesting. Note that the yield was calculated assuming that the oleate ligand on the cluster also acts as a substrate. If one excludes the oleate ligands on the catalyst, the yield is adjusted from 61% to 80% for the reaction with 1.2 equivalents of hexanol. This point is further reinforced by the fact that the recovered cluster catalyst contains oleate ligands on its surface (*vide infra*), suggesting that the oleate ligand is not a substrate.

The substrate scope of the oxo‐cluster catalyst was further explored using sterically hindered alcohols such as 2‐ethylhexanol, benzyl alcohol, and neopentyl alcohol (**Table** **2**) as substrates. While benzyl alcohol converts readily, giving a similar yield to hexanol, 2‐ethyl hexanol, and neopentyl alcohol give a lower yield but still an appreciable conversion. Finally, hafnium oxo clusters (**Hf12**‐oleate; PDF in Figure S4, Supporting Information) exhibit a yield of 57% for the formation of hexyl oleate ester, similar to that of zirconium oxo clusters (61%) (Figure S12, Supporting Information).

**Table 2:** Catalytic esterification of oleic acid with 1.2 equivalents of alcohols, using **Zr12**‐oleate as the catalyst.

Entrya)	Alcohol	Yield [%]	Control [%]	Yield [%]	Control [%]
		3 h		6 h	
(1)	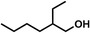	51	25	71	42
(2)	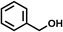	65	19	85	32
(3)	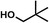	43	23	65	37
(4)	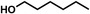	68	28	83	43

a) The catalyst amount is 1 mol% dimer compared to the oleic acid substrate (5 mmol), excluding the oleate ligands of the clusters, thus having slightly different conditions from the ones used in Figure 4. The reaction is conducted at 120 °C with molecular sieves for 3 or 6 h.

### 2.3 Mechanistic Insight into the Active Species

**Figure 5:**
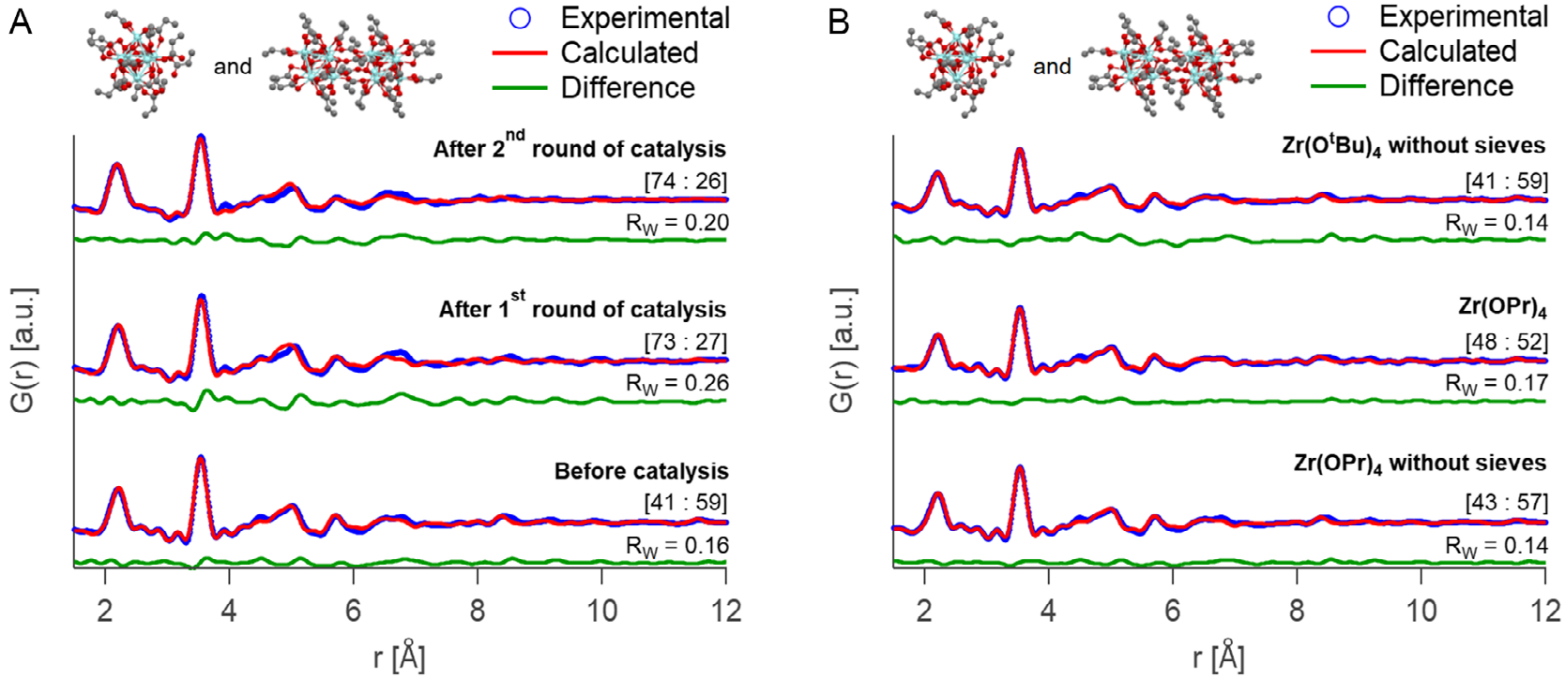
PDF refinement for A) **Zr12**‐oleate cluster before catalysis, and after the first and second round of catalysis, B) and for catalyst recovered after 30 min with and without molecular sieves using ${\text{Zr(OR)}}_{4}$ as the catalyst. The values in square brackets correspond to the ratio of monomer to dimer cluster in the fit. The refinement is performed using both **Zr6**‐ and **Zr12**‐propionate structure obtained from the single crystal structure of ${{\text{[Zr}}_{6}O_{4}{\left(\text{OH}\right)}_{4}{\left(\text{OOCR}\right)}_{12}\text{]}}_{2}$ (CCDC 604529).^[^ ^78^ ^]^ The refined parameters are indicated in Figures S18 and S19, Supporting Information.

Although the clusters are homogeneously dissolved in the reaction mixture, they were recovered after the reaction by precipitation with acetonitrile followed by washing with acetone. Taking the solvent‐free esterification of oleic acid with 1.2 equivalents hexanol as a representative reaction (with 61% yield), the catalyst was recovered (recovery = 46%) and used again in a second reaction (Figure 4). The activity remained the same, suggesting that the catalyst is stable. As shown by thermogravimetric analysis (TGA), the recovered cluster has the same inorganic content as the as‐synthesized cluster (Figure S13, Supporting Information). Also, the nuclear magnetic resonance (NMR) and IR spectra before and after catalysis are the same (Figure S14 and S15, Supporting Information). To gain more precise insight into the structure of the cluster core, we turned to pair distribution function (PDF) analysis.^[^ ^49^ ^]^ **Figure** **5** shows the PDF of the as‐synthesized clusters, and the recovered clusters after the first and second catalytic reactions. The patterns are remarkably alike indicating that the overall ${\text{Zr}}_{6}O_{8}$ cluster structure is retained. However, the dimeric nature of the cluster changes. Before catalysis, the clusters are best described by the dimer structure, while after the reaction, a better refinement was obtained for a monomeric cluster structure (Figure S16, Supporting Information). A dual‐phase refinement was performed combining **Zr6**‐ and **Zr12**‐propionate structure models,^[^ ^78^ ^]^ giving a better fit and providing the ratio between monomer and dimer (Figure 5). This analysis indicated that the structure of the cluster before catalysis was composed of 59% **Zr12** dimer and 41% of **Zr6** monomer. However, after catalysis, the ratio of monomer increases from 41% to 73% after the first cycle and 74% after the second. Most importantly, it can be concluded that the ${\text{Zr}}_{6}O_{8}$ cluster core remains intact and acts as the catalytically active species.

The above mentioned structural conclusions were drawn for reactions where the overall conversion (oleic acid + oleate ligand) does not exceed 76%. Above this conversion, the oleate ligands of the cluster catalyst are converted into ester, which leads to a structural rearrangement or deterioration of the cluster. For example, for a reaction where the conversion was 94%, the cluster structure is severely compromised as indicated by PDF analysis (Figure S20, Supporting Information). When the overall conversion is only slightly above 76%, (e.g., 78%) the ${\text{Zr}}_{6}O_{8}$ cluster structure is still identified in PDF although some structural changes are already present (see Figure S20, Supporting Information).

### 2.4 The Active Species in Homogeneous Catalysis

Zirconium alkoxides were previously employed as homogenous catalysts.^[^ ^79^ ^]^ We hypothesized that such homogenous compounds are simply pre‐catalysts and that they form the **Zr6** clusters in situ. Indeed, we isolated oxo clusters from the esterification reaction of oleic acid by 1.2 equiv hexanol using 12 mol% of ${\text{Zr(OPr)}}_{4}$ or ${\text{Zr(O}}^{t}{\text{Bu)}}_{4}$ (comparable conditions to Table 2). The NMR spectra (Figure S22–S24, Supporting Information) are consistent with oleate‐capped clusters and PDF refinements further confirm the structure of the inorganic cluster core (Figure 5B). The absence or presence of molecular sieves does not change the cluster structure. It is not too surprising that clusters are formed under esterification conditions since esterification is used for cluster synthesis.^[^ ^49^ ^]^ As a final note, ${\text{Zr(O}}^{t}{\text{Bu)}}_{4}$ yielded 63% hexyl oleate ester after 3 h, similar to the yield from cluster catalysts. For ${\text{Zr(OPr)}}_{4}$ the total ester yield was 78%. However, propyl ester was also observed as a side‐product (Figure S21, Supporting Information) due to the higher reactivity of propanol to hexanol. Tert‐butanol does not show any reactivity and hence catalysis using Zr(O^t^Bu)_4_ does not produce side‐products.

## 3 Conclusion

Discrete clusters have superior catalytic activity compared to MOFs and oxide nanocrystals. While oxide nanocrystals have a lower surface area than clusters, MOFs with small pores hamper diffusion, especially for large substrates. Using cluster catalysts, we demonstrated high yields for the esterification of oleic acid with sterically hindered alcohols, in solventless conditions. Through structural studies, aided by X‐ray total scattering and PDF analysis, we confirmed that the oxo clusters are stable during catalysis. The cluster catalysts can be recovered without altering their basic structure or activity. Furthermore, we showed that several homogeneous zirconium and hafnium catalysts transform into oxo clusters during the reaction, thus assigning oxo clusters as the catalytically active species.

## 4 Experimental Section

4.1

4.1.1

##### Materials

Zirconium propoxide (70 w% in 1‐propanol), hafnium butoxide (99%), butyric acid (≥90%), 1,2‐dichlorobenzene (99%, anhydrous), and mesitylene (98%) were provided by Sigma Aldrich and these were stored in a Straus flask upon arrival. Acetic acid (>99%) was purchased from Sigma Aldrich, vacuum transferred, and stored in a Straus flask. Zirconium isopropoxide and zirconium tertbutoxide were synthesized in the Lab.^[^ ^80^ ^]^ Oleic acid (>99%, GC) was bought from TCI chemicals. For cluster synthesis, oleic acid (90%, technical grade) from Sigma Aldrich was used. Benzyl alcohol (anhydrous, 99.8%), 2,2‐dimethyl‐1‐propanol (99%), and 2‐ethyl‐1‐hexanol (≥99.6%) were bought from Sigma Aldrich and used without any further purification. 1‐Hexanol (99%, anhydrous) was brought from Acros. Acetone and dichloromethane (DCM) were bought from Biosolve and used without any further purification. Acetonitrile (ACN) HPLC grade was bought from VWR chemicals and used without any further purification. Molecular sieves, 3 Å (beads, 8–12 mesh) were purchased from Sigma Aldrich and were activated under vacuum before use.

##### Synthesis of ${\text{ZrO}}_{2}$ Nanocrystals with Oleate Ligands

${\text{ZrO}}_{2}$ nanocrystals were synthesized according to Garnweitner et al. using zirconium isopropoxide isopropanol complex (6.6 g, 17 mmol, 1 eq) and benzyl alcohol (60 mL, 580 mmol, 34 eq) at 210 °C for 2 days.^[^ ^73^ ^]^ After the reaction, a white powder was isolated via centrifugation. The powder was washed three times with diethyl ether and was then dispersed in 40 mL toluene for functionalizing with 2.4 mL oleic acid (99%). Thus formed nanocrystals were precipitated using 30 mL acetone. Finally, the purified nanocrystals were dispersed in 20 mL of toluene and stored in a fridge. TGA showed the presence of 65% inorganic content and a yield of 43% was obtained.

##### Synthesis of UiO‐66

Synthesis was done with slight modifications to previous reports.^[^ ^71^, ^72^ ^]^ ${\text{ZrCl}}_{4}$ (3.5 g, 15 mmol) and benzene‐1, 4‐dicarboxylic acid (2.5 g, 15 mmol) were mixed with 155 mL of dimethyl formamide (DMF)and 1.5 mL of hydrochloric acid (HCl) (37%). The reaction was done in a 1 L pressure bottle at 120 °C for 24 h. After 24 h, a white precipitate was formed. The white precipitate was collected via centrifugation. This was washed by adding acetone and shaking for 2 days. The solvent was changed four times within the 2 days of washing. The powder was collected using centrifugation at 10000 rpm for 10 min. It was dried in air at 70 °C for 4 h followed by activation at 110 °C for 20 h, and stored in a desiccator.

##### Synthesis of Zr12 oxo Clusters

All oxo clusters were synthesized and purified according to our previous report.^[^ ^49^ ^]^

##### Catalytic Experiments: Ethyl oleate using Zr12 Cluster as Catalyst

The standard catalytic reaction used 1 mol% of **Zr12** cluster as catalyst (0.096 mmol Zr, 64.9 mg cluster). The 1 mol% is calculated with respect to the limiting reagent (oleic acid + oleate ligand = 0.8 mmol). The catalyst was transferred to an 8 mL GC vial, together with oleic acid (0.608 mmol, 192 μL). Furthermore, four equivalents of ethanol (3.2 mmol, 186.8 μL) and 3.6 mL of either *o*‐dichlorobenzene (*o*‐DBC) or mesitylene were added, together with 100 mg of 3 Å molecular sieves. The total solution was 4 mL and the final oleate/oleic acid concentration was 0.2 M. The solution was stirred on a heating block at 120 °C for 12 h, after which the yield was determined by ^1^H NMR.

##### Catalytic Experiments: Ethyl oleate using 5.6 nm ${\text{ZrO}}_{2}$ Nanocrystals as Catalyst

Oleic acid (0.78 mmol, 246 μL) was mixed with 4 eq of EtOH (with respect to 0.8 mmol of oleic acid + oleate ligand) and 3 Å molecular sieves and made up to 4 mL using *o*‐DCB as the solvent. To this, was added, 0.096 mmol ${\text{ZrO}}_{2}$ (18.15 mg) which corresponds to the amount of Zr present in 1 mol% of the **Zr12** cluster. The reaction was stirred in an 8 mL GC vial at 120 °C for 12 h, after which the yield was determined by ^1^H NMR.

##### Catalytic Experiments: Ethyl oleate using UiO‐66 as Catalyst

Oleic acid (0.8 mmol, 252 μL) was mixed with 4 eq of EtOH and 3 Å molecular sieves and made up to 4 mL using *o*‐DCB as the solvent. To this, was added, 24.34 mg of MOF UiO‐66 (0.096 mmol of Zr). The reaction was stirred in an 8 mL GC vial at 120 °C for 12 h, after which the yield was determined by ^1^H NMR.

##### Catalytic Experiments: Hexyl oleate using Zr12‐Oleate as Catalyst

3.8 mmol of oleic acid (1.199 mL) was mixed with 4 eq or 1.2 eq of EtOH and 3 Å molecular sieves. To this, was added, 1 mol% of the Zr12 oleate cluster (405.7 mg)(the total amount of oleic acid and oleate is 5 mmol). The reaction was stirred in an 8 mL GC vial at 120 °C. Aliquotes were taken after 3 and 6 h and the reaction progress was calculated based on the ^1^H NMR data. The catalyst was then recovered by precipitation using acetonitrile and washing with acetone (recovery = 46%).

##### Catalytic Experiments: 2‐Ethylhexyl, Benzyl, and Neopentyl Oleate using Zr12 Oleate as Catalyst

The reaction was done similarly to the aforementioned procedure using 5 mmol of oleic acid and 1.2 equivalent of the respective alcohol.

##### Catalytic Experiments: Hexyl oleate using Homogeneous Catalysts

5 mmol of oleic acid (1.578 mL) is mixed with 1.2 eq of hexanol (753.1 μL) and 3 Å molecular sieves (omitted in some reactions). To this, is added, 12 mol% (0.6 mmol) of ${\text{Zr(OPr)}}_{4}$ (268.9 μL), or ${\text{Zr(O}}^{t}{\text{Bu)}}_{4}$ (233.7 μL). The reaction is stirred in a GC vial for 30 min or 3 h and analyzed via ^1^H NMR. The catalyst is then recovered by precipitation using acetonitrile and washing with acetone.

##### General Instrumentation

The fourier‐transform infrared (FTIR) analysis was done on Perkin Elmer spectrum 2 ATR‐FTIR with a diamond crystal. The thermogravimetric analysis (TGA) was performed on a TGA5500 (TA instruments) instrument. All TGA measurements were done under air (oxidizing atmosphere). The samples were heated to 900 °C at a ramping rate of 5 °C min^−1^. At the end, an isotherm of 15 min is given to ensure that all the organics are burned out. HR‐TEM imaging was carried out in JEOL JEM‐F200 operated in the TEM‐mode at a beam energy of 200 kV.

##### General Instrumentation: NMR Spectroscopy

NMR measurements were recorded at 298 K on Bruker UltraShield 500 spectrometer. To track the esterification yield, NMR spectroscopy was employed as the primary technique. The *α* proton of the ester formed during oleate esterification shows a distinct peak in the NMR. The integral of this peak was compared with the alkene resonance at 5.4 ppm to determine the percentage conversion. The delay time for the proton NMR was set optimized to reduce the error in the percentage conversion calculation using NMR. A delay time of 10 and 30 s shows almost similar results. Therefore the delay time for further studies was fixed at 10 s.

##### General Instrumentation: Structural Characterization with X‐ray Total Scattering Experiments

X‐ray total scattering data were collected at beamline 28‐ID‐2 (XPD) at National Synchrotron Light Source II (NSLS‐II), Brookhaven National Laboratory, USA or at beamline P21.1 at PETRA III/DESY in Hamburg, Germany. Measurements were carried out at room temperature on samples prepared in 1 mm polyamide kapton tubes in rapid acquisition mode using a large‐area 2D PerkinElmer detector (2048 × 2048 pixels, 200 μm × 200 μm pixel size) with a sample‐to‐detector distance of 267 mm (28‐ID‐2) or 350 mm (P21.1). The incident wavelength of the X‐rays was *λ* = 0.1665 Å (28‐ID‐2) and *λ* = 0.1222 Å (P21.1) and the measurement exposure time was 600 s. To calibrate the experimental setup, a ${\text{CeO}}_{2}$ standard was used while the scattering pattern of pure oleic acid or of the empty kapton was used as background, in accordance with experimental conditions. The data were integrated using pyFAI^[^ ^81^ ^]^ and the PDF was obtained using xPDFSuite^[^ ^82^ ^]^ with PDFgetX3 with *Q*_max_ = 21.5, *Q*_min_ = 0.8 and *R*_poly_ = 0.99. The chemical composition of the cluster (${\text{Zr}}_{12}O_{64}C_{24}H_{80}$) was used to reduce the data. DiffpyCMI^[^ ^83^ ^]^ was used to fit the data by refining scale factor (one per each phase in case of the dual phase fitting), isotropic atomic displacement parameters (Uiso), and delta2 (coefficient for the 1/r2 contribution to peak sharpening).

## Conflict of Interest

The authors declare no conflict of interest.

## Supporting information

Supplementary Material

## Data Availability

The data that support the findings of this study are available in the supplementary material of this article. The data is freely available via https://doi.org/10.5281/zenodo.13782982.
