# Supplementary material for: The Central Role of Oxo Clusters in Zirconium‐Based Esterification Catalysis
Source: Small Sci. 2024 Sep 23;5(1):2400369. doi: 10.1002/smsc.202400369 (PMC11935207; doi:10.1002/smsc.202400369)
Supplement: Supplementary file 1 — Supplementary Material [file SMSC-5-2400369-s001.pdf]

Supporting Information: The central role of oxo clusters in zirconium-based esterification catalysis

Jikson Pulparayil Mathew,† Carlotta Seno,† Mohit Jaiswal,‡ Charlotte Simms,¶ Nico Reichholf,† Dietger Van den Eynden,† Tatjana N. Parac-Vogt,¶ and Jonathan De Roo*,†

†*Department of Chemistry, University of Basel, Mattenstrasse 24a, 4058 Basel, Switzerland*

‡*Department of Chemical Sciences, Indian Institute of Science Education and Research (IISER)-Mohali, Mohali, SAS Nagar, Punjab, 140306, India*

¶*Department of Chemistry, KU Leuven, Celestijnenlaan 200F, 3001 Leuven, Belgium*

E-mail: Jonathan.DeRoo@unibas.ch

Catalyst characterization

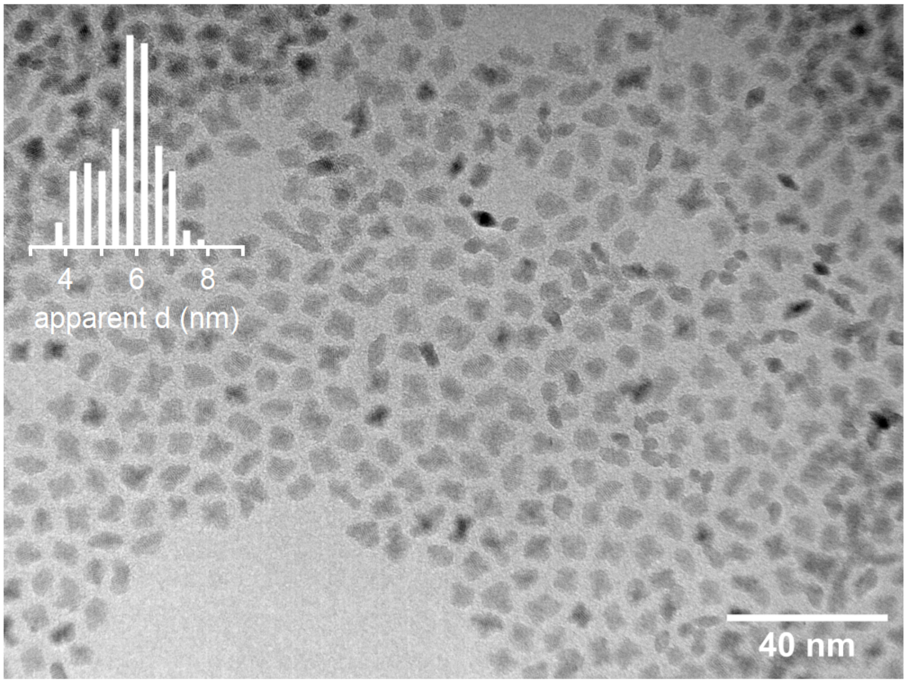

Figure S1: TEM image of ZrO_2 synthesized using autoclave and oleic acid as the ligand. The histogram in the inset shows the size distribution of the nanocrystal.

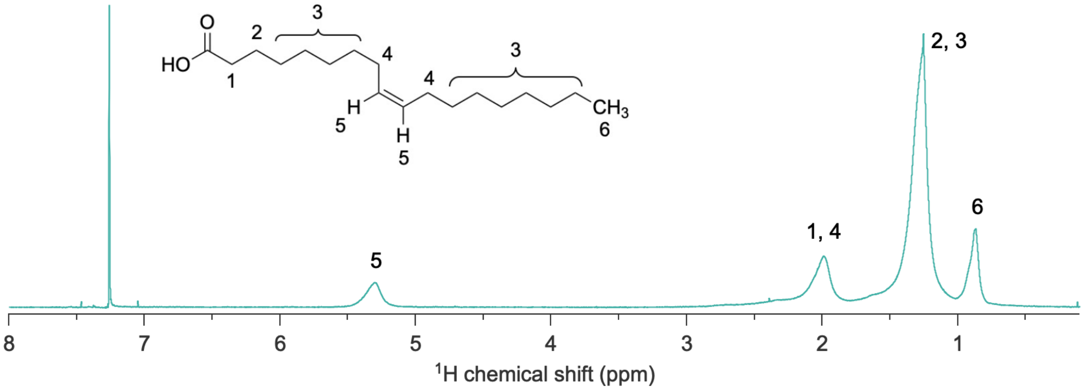

Figure S2: ^{1}H NMR spectra of ZrO_2 nanocrystal to verify the presence of oleate on the surface.

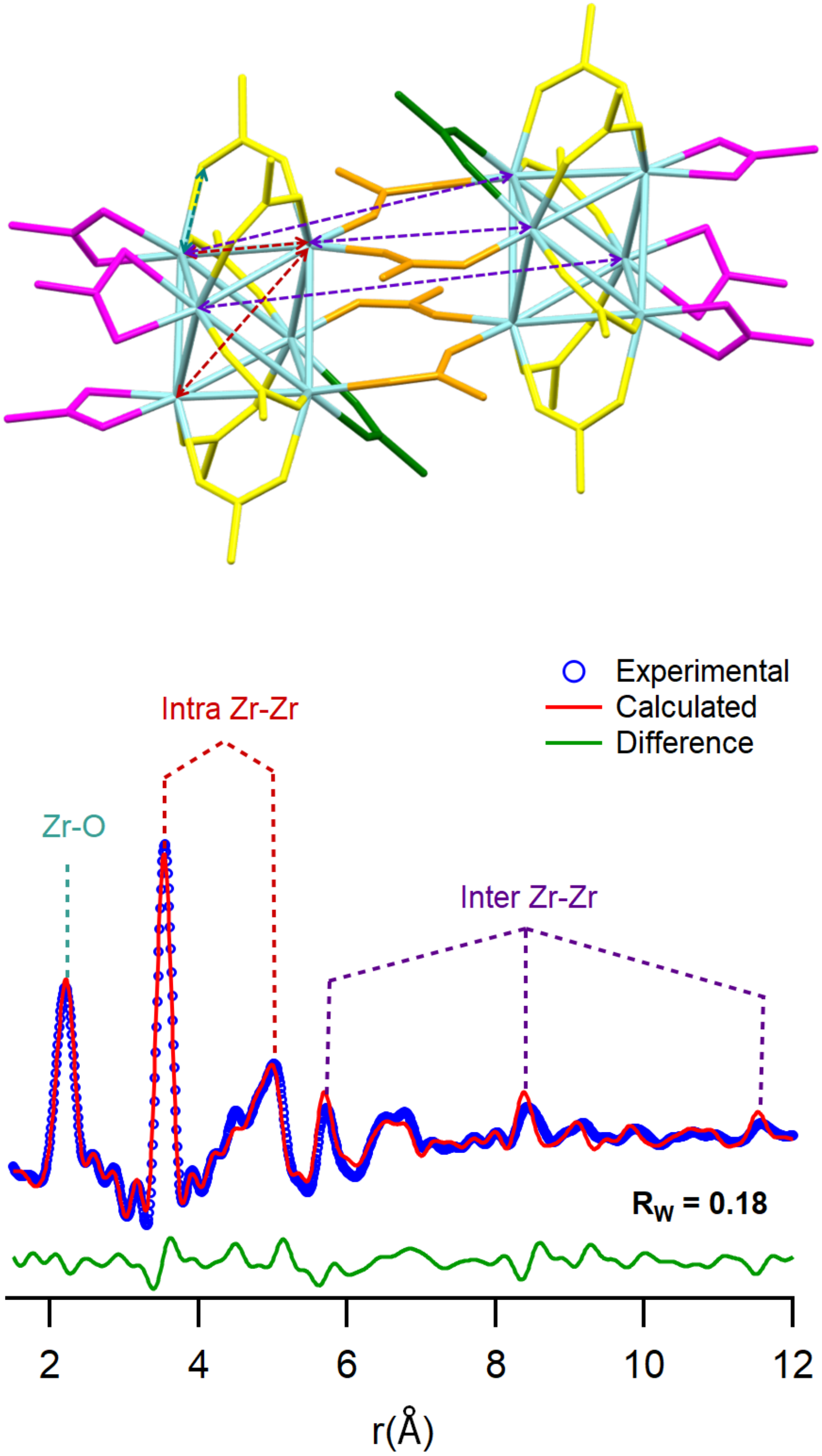

Figure S3: PDF of **Zr12**-oleate. The fitting was done using **Zr12**-propionate structure model obtained from the single crystal structure of $[Zr_6O_4(OH)_4(OOCR)_{12}]_2$ (CCDC 604529).[S1] The refined parameters were indicated in Figure S5.

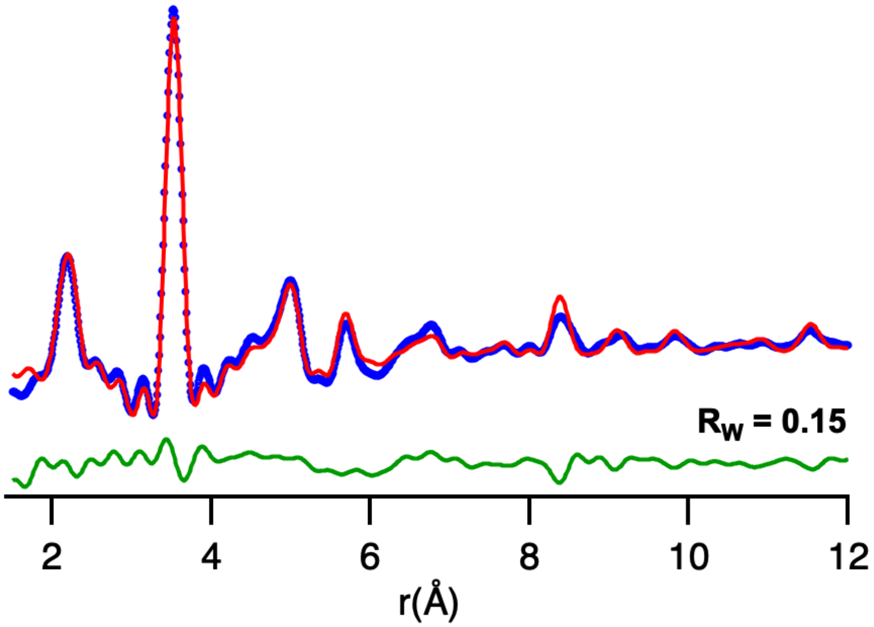

Figure S4: PDF refinement for **Hf12**-oleate. The fitting was done using **Hf12**-propionate structure model obtained from the single crystal structure of $[Zr_6O_4(OH)_4(OOCR)_{12}]_2$ (CCDC 604529).[S1] The refined parameters were indicated in Figure S5.

Uiso_C_G1	9.47434859e-03	+/-	5.08513853e-03
Uiso_O_G1	1.02119368e-02	+/-	4.01615934e-03
Uiso_Zr_G1	3.05410861e-03	+/-	3.87119104e-04
delta2	3.64347332e+00	+/-	6.23471804e-01
scale	6.71679028e-01	+/-	2.23573666e-02

(a) **Zr12**-oleate before catalysis fitted with **Zr12**-propionate.

Uiso_C_G1	2.35468704e-02	+/-	4.63489720e-02
Uiso_Hf_G1	4.68910536e-03	+/-	9.59367185e-04
Uiso_O_G1	3.55089955e-02	+/-	4.60132128e-02
delta2	4.53869375e+00	+/-	5.42964567e-01
scale	1.97443631e-01	+/-	1.41846411e-02

(b) **Hf12**-oleate before catalysis fitted with **Hf12**-propionate.

Figure S5: Refined parameters for PDF fitting in Figure S3 and Figure S4.

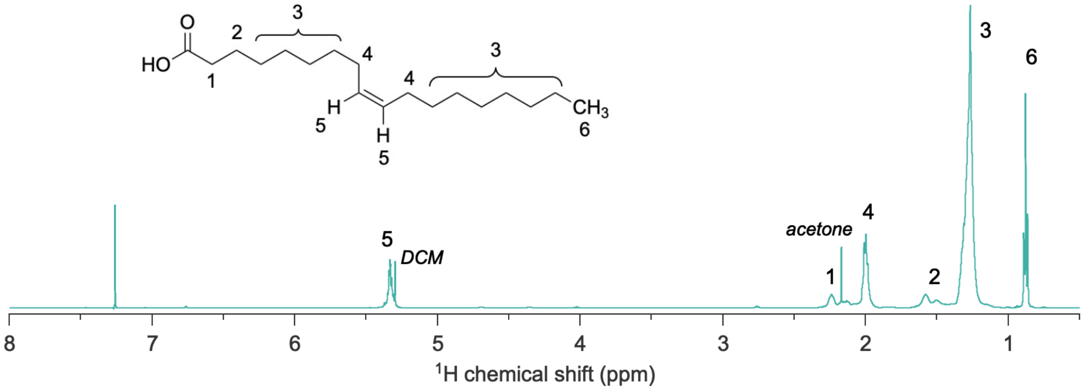

Figure S6: ^{1}H NMR spectrum of **Zr12**-oleate cluster to verify the presence of oleate on the surface.

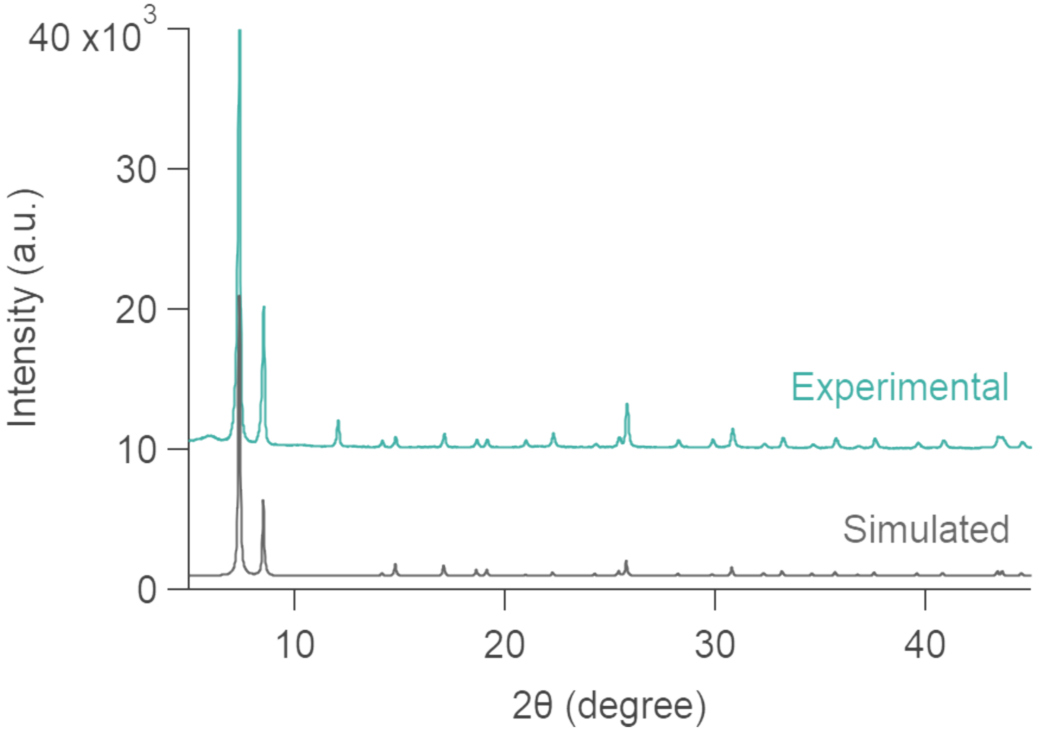

Figure S7: pXRD of the synthesized UiO-66. The simulated data is based on the single crystal data from CCDC 837796.[S2]

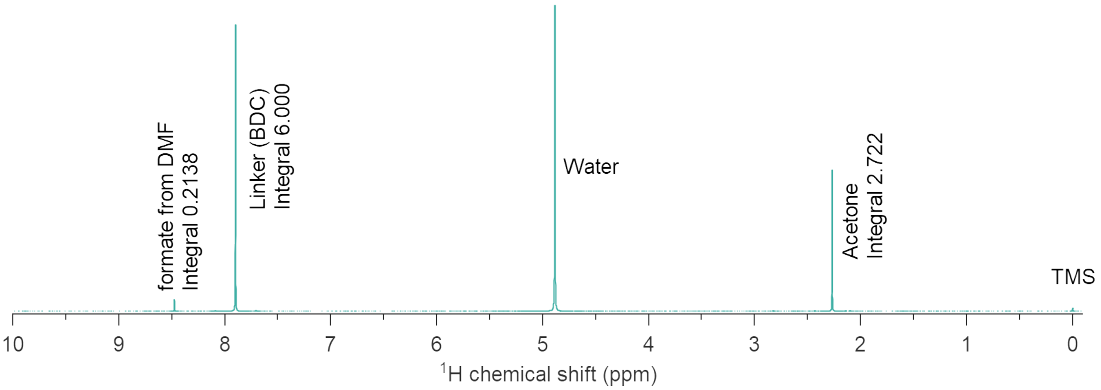

Figure S8: ^{1}H NMR spectra of UiO-66 after digestion of the MOF using 1M NaOH.

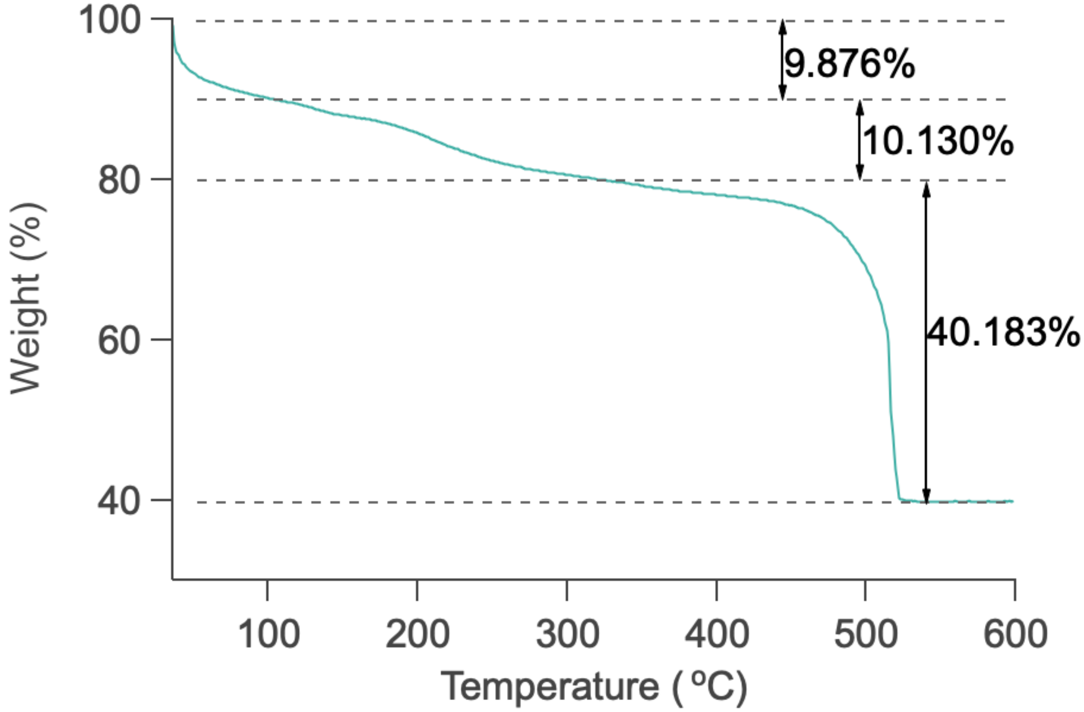

Figure S9: TGA of the synthesised UiO-66

At the plateau between 300 and 500 °C, the UiO-66 dehydrates, and the modulators were removed which were seen in the ^{1}H NMR spectra (Figure S8). This gives a minimal formula of $ZrO(C_6H_4C_2O_4)$ per Zr atom. Assuming the end product of TGA to be ZrO_2, we determined that the final inorganic content in an ideal UiO-66 corresponds to 45.4%. This means that the linker amount is 54.6% in an ideal UiO-66 as described by L. Valenzano *et al.*[S2,S3] The

amount of linker in the synthesised UiO-66 was 40.183% (Figure S9). This suggests that 26.4% linkers were missing per formula unit.

Turnover number (TON) calculation

The concentration of oleic acid in the reaction mixture = 200 mM [this includes the oleic acid present on the catalyst's surface also].

TON for nanocrystal (NCs)

The reaction progress using NC as a catalyst for ethyl oleate formation after 12 hrs = 16.24 %. Reaction progress after subtracting the reaction progress from the control = 4.47 % (16.24 - 11.77).

$$\begin{aligned} Concentration\ of\ \mathrm{ZrO_2} &= \frac{mmols\ of\ \mathrm{ZrO_2}}{volume\ of\ reaction\ mixture} \\ &= \frac{0.096}{4} \\ &= 0.024M \\ &= 24mM \end{aligned} \tag{1}$$

$$Number\ of\ active\ sites = Number\ of\ ligand\ present\ on\ the\ nanoparticle\ surface \tag{2}$$

From ERETIC measurements, we know that 33.4 mM of NCs contain 6.479 mmol/L of ligand. Therefore, 24 mM of NC will contain 4.66 mmol/L of ligand.

$$
\begin{aligned}
TON &= \frac{[products]}{[active\ sites]} \\
&= \frac{\%\ yield \times [oleic\ acid]}{[active\ sites]} \\
&= \frac{0.0447 \times 200}{4.66} \\
&= 1.9
\end{aligned}
\tag{3}
$$

TON for Zr-12 oleate cluster

The reaction progress using cluster as a catalyst for ethyl oleate formation after 12 hrs = 89.6 %. Reaction progress after subtracting the reaction progress from the control = 77.83 % (89.6 - 11.77).

$$
\begin{aligned}
Number\ of\ active\ sites &= Number\ of\ zirconium\ atoms. \\
&= 0.008 \times 12 \\
&= 0.096 mmol \\
&= \frac{0.096}{4} M \\
&= 24 mM
\end{aligned}
\tag{4}
$$

$$
\begin{aligned}
TON &= \frac{[products]}{[active\ sites]} \\
&= \frac{\%\ yield \times [oleic\ acid]}{[active\ sites]} \\
&= \frac{0.7783 \times 200}{24} \\
&= 6.5
\end{aligned}
\tag{5}
$$

Catalytic reaction analysis

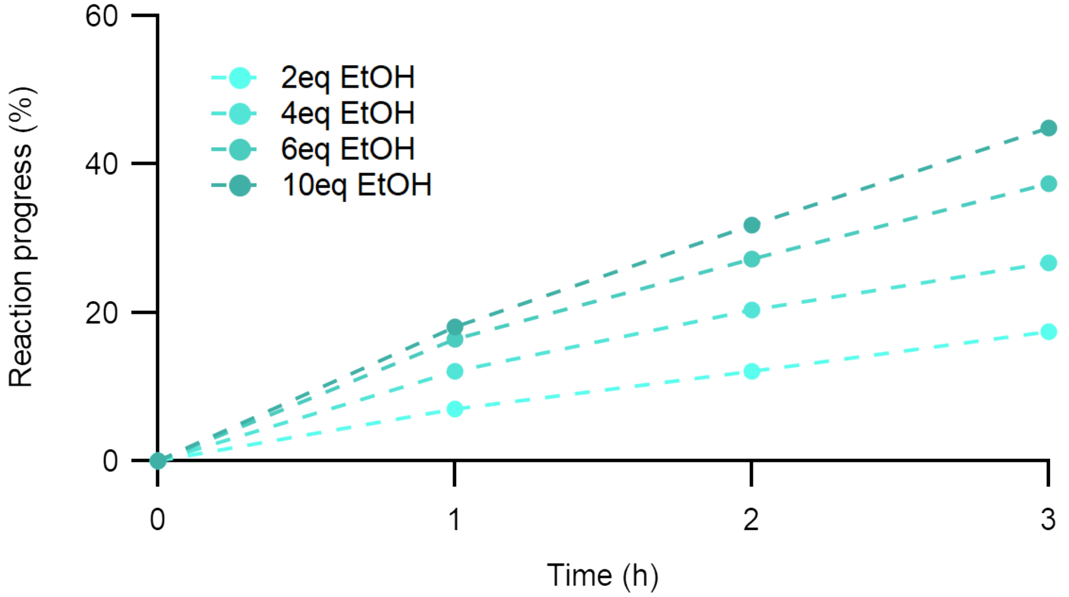

Figure S10: Ethanol equivalent optimization of ethyl oleate esterification using **Zr12**-oleate. The reaction was done in *o*-DCB using 1 mol% cluster, 200 mM OA, EtOH, and 3 Å molecular sieves in 4 mL solution for 3 h at 120 °C. Aliquots were taken every 1 h.

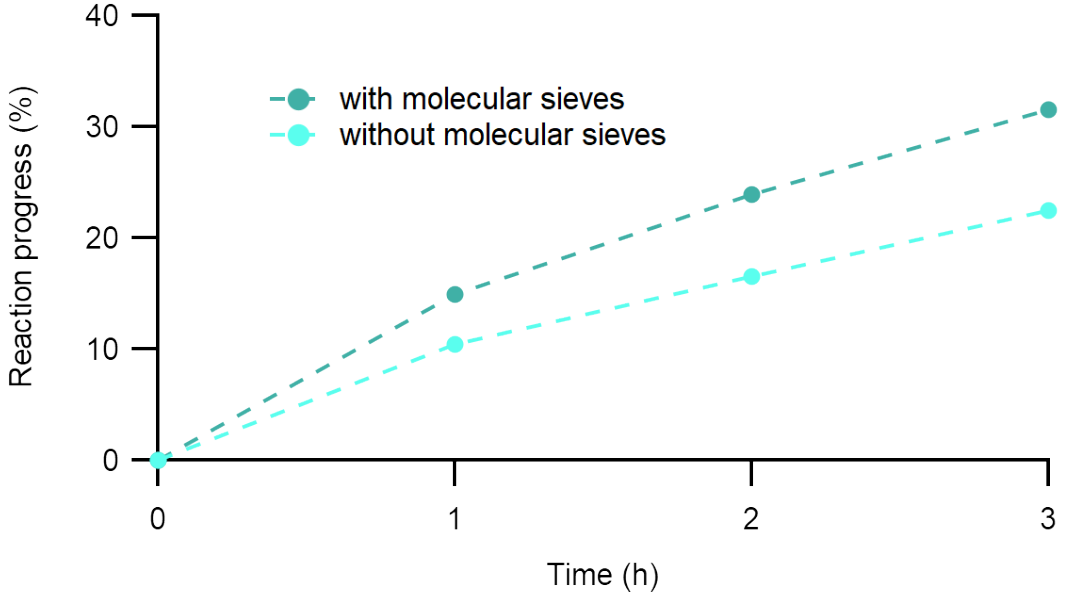

Figure S11: Water dependence study of ethyl oleate esterification using **Zr12**-oleate. The reaction was done in *o*-DCB using 1 mol% cluster, 200 mM OA, and 4 eq EtOH in 4 mL solution for 3 h at 120 °C. Aliquots were taken every 1 h. 3 Å molecular sieves were used in this study for absorbing the water that was formed in this reaction.

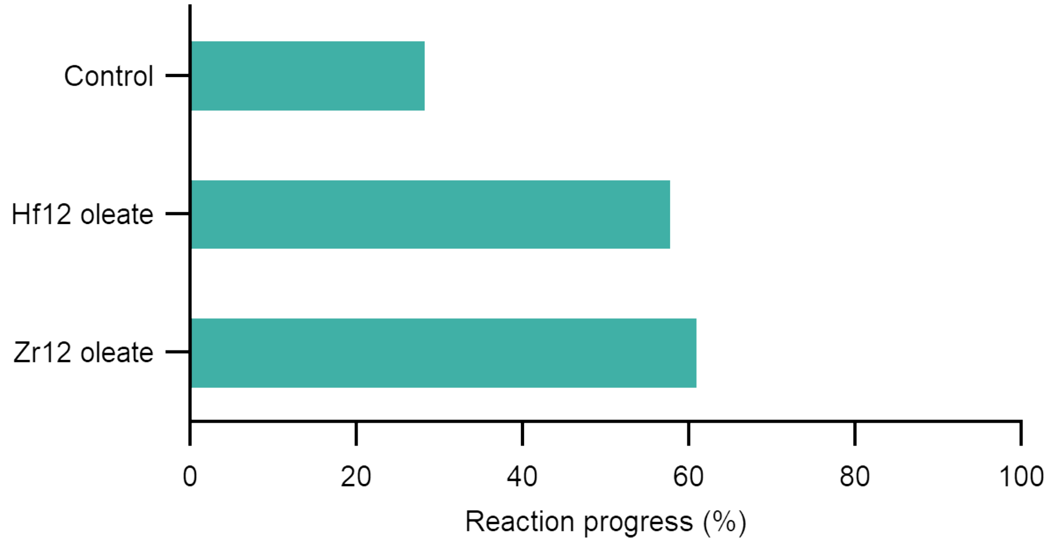

Figure S12: Comparison of catalytic activity of zirconium and hafnium cluster. The reaction was done in *o*-DCB using 1 mol% cluster, 200 mM OA, 4 eq EtOH, and 3 Å molecular sieves in 4 mL solution for 3 h at 120 °C.

Cluster recovery and mechanism

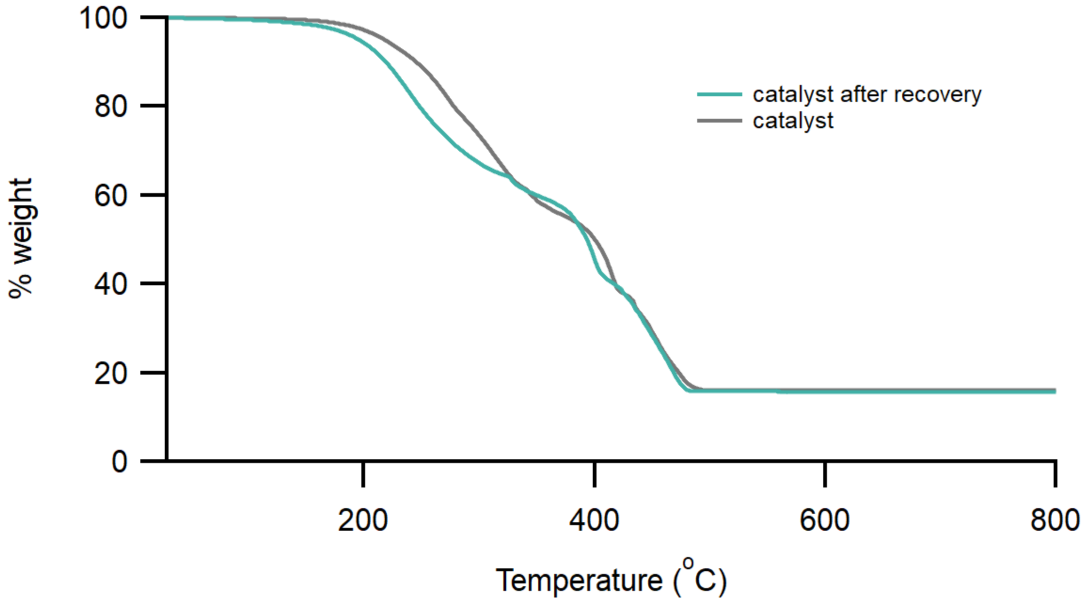

Figure S13: TGA of **Zr12**-oleate cluster recovered after esterification reaction. The TGA of the **Zr12**-oleate before esterification is given in grey.

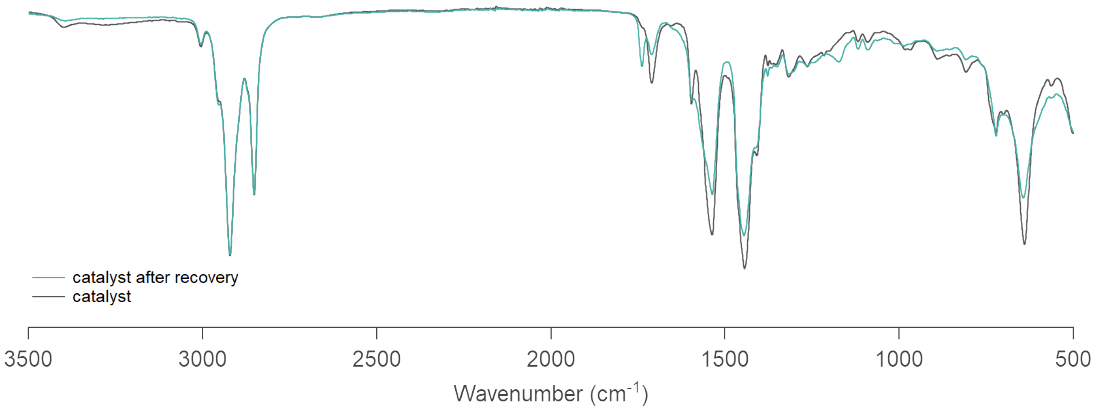

Figure S14: FTIR spectra of **Zr12**-oleate cluster recovered after esterification reaction. The FTIR of the **Zr12**-oleate before esterification is given in grey spectra.

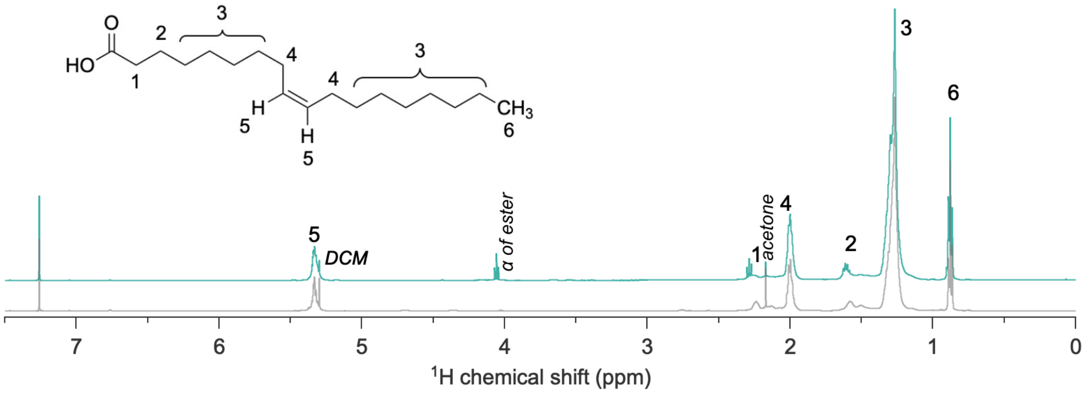

Figure S15: ^{1}H NMR spectra of **Zr12**-oleate cluster recovered after esterification reaction. The spectra of the **Zr12**-oleate before esterification is given in grey spectra. The spectrum of the recovered cluster shows the presence of ester that is difficult to remove during purification (sharp peaks).

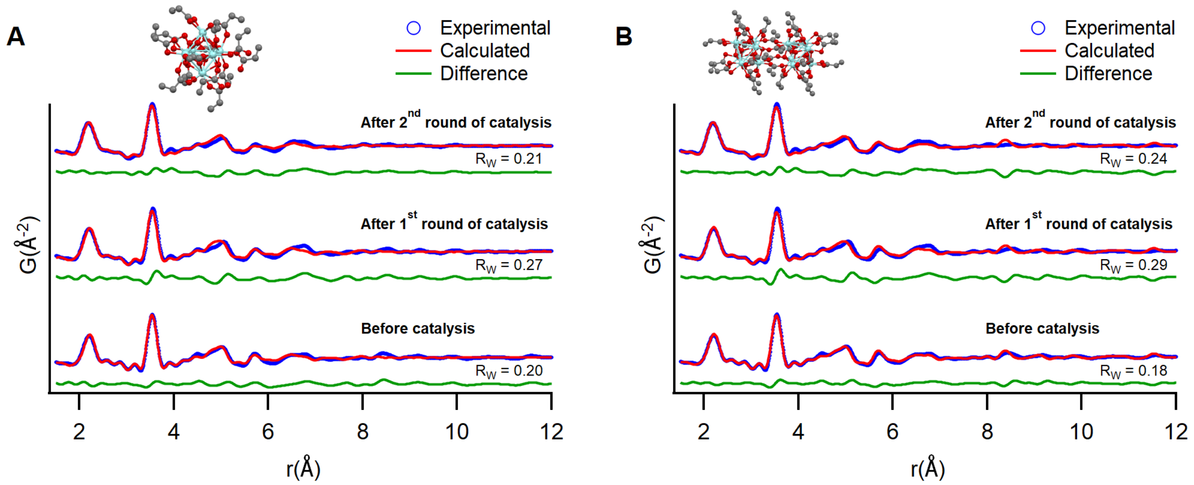

Figure S16: PDF refinement for the **Zr12**-oleate cluster before catalysis, and after the first and second round of catalysis. The refinement is performed using A) **Zr6**-propionate and B) **Zr12**-propionate structure model obtained from the single crystal structure of $[Zr_6O_4(OH)_4(OOCR)_{12}]_2$ (CCDC 604529).[S1] The refined parameters are indicated in Figure S17.

```
Uiso_C_G1    6.82737558e-03 +/- 3.85347131e-03
Uiso_O_G1    3.17415826e-03 +/- 1.76500800e-03
Uiso_Zr_G1   2.33375201e-03 +/- 4.27268454e-04
delta2       6.51254303e-01 +/- 1.96977592e+00
scale        6.95116336e-01 +/- 2.29834943e-02
```

(a) **Zr12** before catalysis fitted with **Zr6**-propionate.

```
Uiso_C_G1    9.47434859e-03 +/- 5.08513853e-03
Uiso_O_G1    1.02119368e-02 +/- 4.01615934e-03
Uiso_Zr_G1   3.05410861e-03 +/- 3.87119104e-04
delta2       3.64347332e+00 +/- 6.23471804e-01
scale        6.71679028e-01 +/- 2.23573666e-02
```

(b) **Zr12**-oleate before catalysis fitted with **Zr12**-propionate.

```
Uiso_C_G1    2.77691565e-03 +/- 2.73852362e-03
Uiso_O_G1    4.16570333e-03 +/- 2.22551466e-03
Uiso_Zr_G1   3.70491213e-03 +/- 5.00487017e-04
delta2       2.07698310e+00 +/- 1.22649866e+00
scale        7.45615889e-01 +/- 2.40721144e-02
```

(c) **Zr12**-oleate after first round of catalysis fitted with **Zr6**-propionate.

```
Uiso_C_G1    5.84312580e-03 +/- 4.01259386e-03
Uiso_O_G1    1.33520845e-02 +/- 4.62034298e-03
Uiso_Zr_G1   4.56781813e-03 +/- 4.64114838e-04
delta2       3.95537350e+00 +/- 4.12309630e-01
scale        7.18957857e-01 +/- 2.37768781e-02
```

(d) **Zr12**-oleate after first round of catalysis fitted with **Zr12**-propionate.

```
Uiso_C_G1    1.45866838e-02 +/- 7.72573945e-03
Uiso_O_G1    2.45400955e-02 +/- 6.01397607e-03
Uiso_Zr_G1   4.56494220e-03 +/- 4.87679586e-04
delta2       4.18326437e+00 +/- 2.40585820e-01
scale        7.50634435e-01 +/- 2.50038844e-02
```

(e) **Zr12**-oleate after second round of catalysis fitted with **Zr6**-propionate.

```
Uiso_C_G1    2.01504365e-02 +/- 1.00461087e-02
Uiso_O_G1    3.36834606e-02 +/- 8.45547284e-03
Uiso_Zr_G1   5.10000505e-03 +/- 5.15099769e-04
delta2       4.33238600e+00 +/- 1.77555681e-01
scale        7.33047436e-01 +/- 2.37347903e-02
```

(f) **Zr12**-oleate after second round of catalysis fitted with **Zr12**-propionate.

Figure S17: Refined parameters for PDF fitting in Figure S16

.

```
C_Uiso         7.58245450e-03 +/- 4.28901348e-03
Delta2         2.86348228e+00 +/- 1.01451093e+00
O_Uiso         7.00472792e-03 +/- 3.19572855e-03
Zr_Uiso        2.76489188e-03 +/- 3.98121275e-04
scale_dimer    4.01812530e-01 +/- 8.87300158e-02
scale_monomer  2.80374715e-01 +/- 9.11211793e-02
```

(a) **Zr12**-oleate before catalysis.

```
C_Uiso         3.38087773e-03 +/- 3.01256239e-03
Delta2         2.71228566e+00 +/- 9.85315763e-01
O_Uiso         5.86109186e-03 +/- 2.87421386e-03
Zr_Uiso        3.96716675e-03 +/- 4.97259966e-04
scale_dimer    1.96091281e-01 +/- 9.60774943e-02
scale_monomer  5.40682427e-01 +/- 9.94389588e-02
```

(b) **Zr12**-oleate recovered after the first cycle of catalysis.

```
C_Uiso         1.58049374e-02 +/- 8.12948143e-03
Delta2         4.23528975e+00 +/- 2.11281122e-01
O_Uiso         2.83519006e-02 +/- 6.88051793e-03
Zr_Uiso        4.66509716e-03 +/- 4.95725951e-04
scale_dimer    1.94963381e-01 +/- 9.91704729e-02
scale_monomer  5.49164349e-01 +/- 1.01581956e-01
```

(c) **Zr12**-oleate recovered after the second cycle of catalysis.

Figure S18: Refined parameters for PDF fitting with both Zr6 and Zr12 propionate clusters in Figure 5A.

C_Uiso	9.07045789e-03	+/-	5.84285133e-03
Delta2	3.14265385e+00	+/-	1.09450546e+00
O_Uiso	7.42669378e-03	+/-	3.95980103e-03
Zr_Uiso	2.62357176e-03	+/-	4.61338276e-04
scale_dimer	3.22798867e-01	+/-	8.74913728e-02
scale_monomer	2.43565556e-01	+/-	8.96677640e-02

(a) $Zr(OPr)_4$ without sieves.

C_Uiso	2.01433130e-02	+/-	1.66038495e-02
Delta2	2.00211228e+00	+/-	1.41773448e+00
O_Uiso	5.24759579e-03	+/-	3.41311477e-03
Zr_Uiso	2.23024691e-03	+/-	5.63067229e-04
scale_dimer	2.09806975e-01	+/-	8.38378883e-02
scale_monomer	1.94553427e-01	+/-	8.57106060e-02

(b) $Zr(OPr)_4$ with sieves.

C_Uiso	1.05520789e-02	+/-	3.82414566e-03
Delta2	2.73078341e+00	+/-	6.79497034e-01
O_Uiso	8.37038576e-03	+/-	2.57961363e-03
Zr_Uiso	3.18904880e-03	+/-	2.97995112e-04
scale_dimer	5.96685004e-01	+/-	9.29531300e-02
scale_monomer	4.19255514e-01	+/-	9.49846054e-02

(c) $Zr(O^tBu)_4$ without sieves.

Figure S19: Refined parameters for PDF fitting with both Zr6 and Zr12 propionate clusters in Figure 5B.

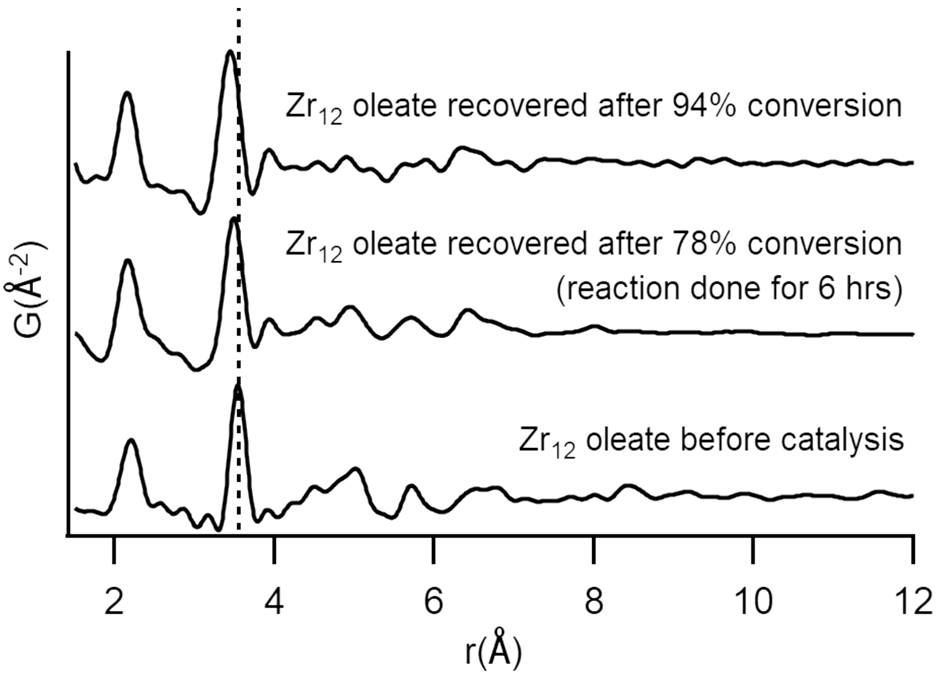

Figure S20: PDF model-free analysis of **Zr12**-oleate cluster before and after catalysis at extreme conditions. In the plot on the middle **Zr12**-oleate cluster was recovered after pushing the reaction progress to 78%. In the top plot, the synthesis of hexyloleate was initially done at 120 °C for 3 hrs and then increased to 180 °C for the next 5 hrs. 80 eq of hexanol was used so that the concentration of oleic acid remained 200 mM. The recovered species do not have the same structure as the initial one, the catalytic conditions destroyed the **Zr12** cluster.

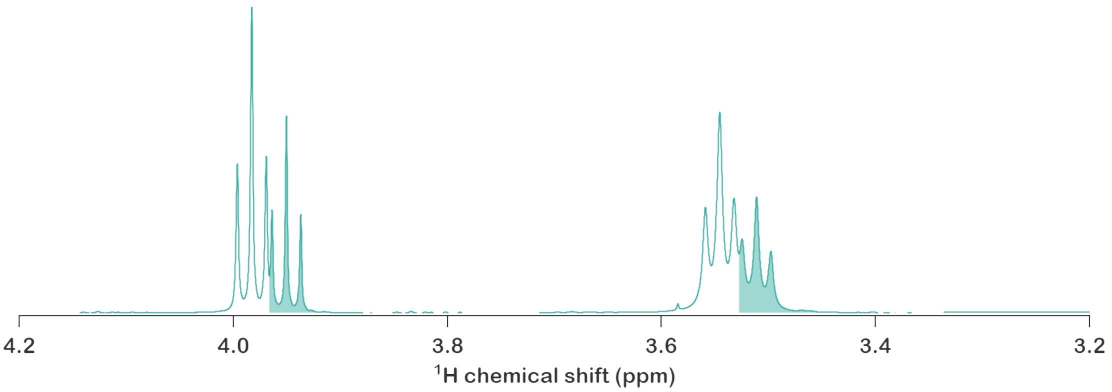

Figure S21: ^{1}H NMR spectra of hexyl oleate formation using $Zr(OPr)_4$ as the catalyst. The spectra show the formation of propyl oleate along with hexyl oleate. The shaded region corresponds to the propyl oleate that is formed, and the un-shaded region represents the hexyl oleate which is the main product.

Table S1: Reaction progress of hexyl oleate formation after 30 min using precursor used for cluster formation

Entry	Catalyst	Sieves	Reaction progress
(1)	$Zr(OPr)_4$	Yes	32%
(2)	$Zr(OPr)_4$	No	24%
(4)	$Zr(OtBu)_4$	No	21%

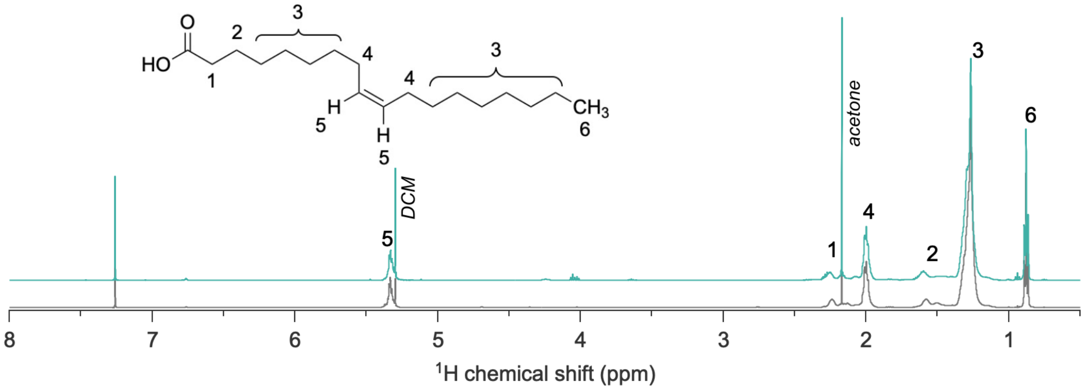

Figure S22: ${}^{1}H$ NMR spectra of the catalyst recovered after the formation of hexyl oleate. The reaction uses $Zr(OPr)_4$ as the catalyst for 30 min in the presence of molecular sieves. Grey spectrum corresponds to the ${}^{1}H$ NMR spectra of **Zr12**-oleate cluster.

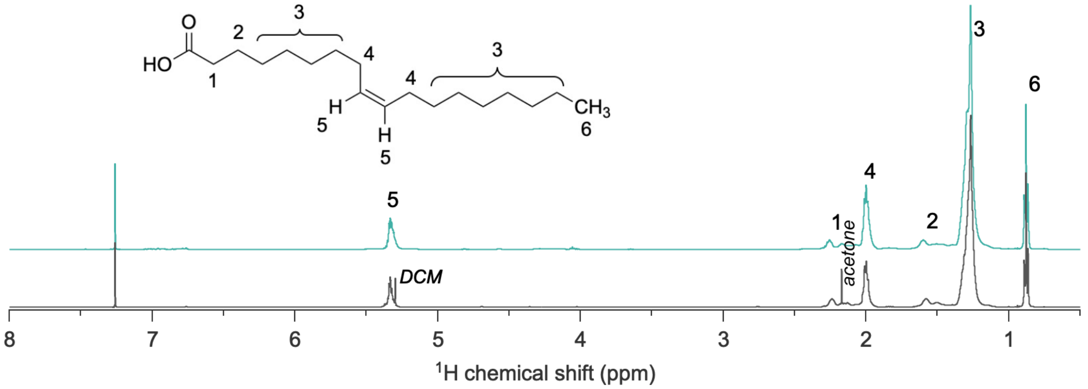

Figure S23: ${}^{1}H$ NMR spectra of the catalyst recovered after the formation of hexyl oleate. The reaction uses $Zr(OPr)_4$ as the catalyst for 30 min in the absence of molecular sieves. Grey spectrum corresponds to the ${}^{1}H$ NMR spectra of **Zr12**-oleate cluster.

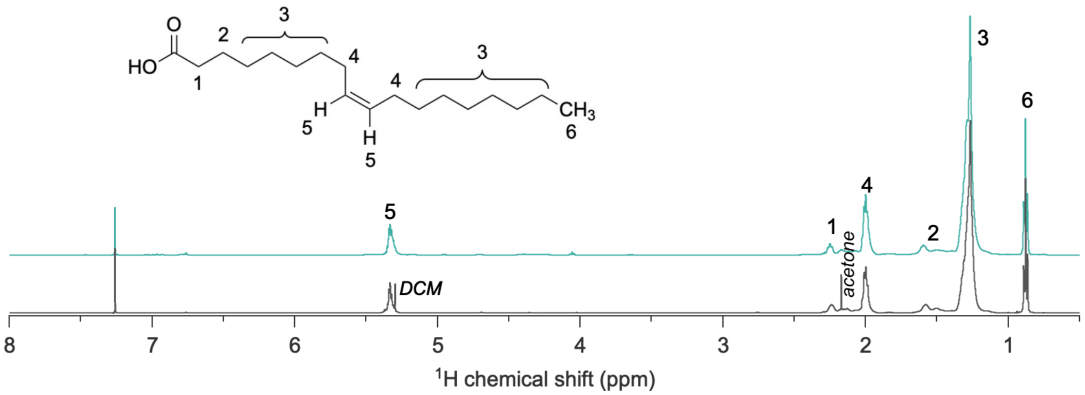

Figure S24: ^{1}H NMR spectra of the catalyst recovered after the formation of hexyl oleate. The reaction uses $Zr(O^{t}Bu)_4$ as the catalyst for 30 min in the absence of molecular sieves. Grey spectrum corresponds to the ^{1}H NMR spectra of **Zr12**-oleate cluster.

References

(S1) Puchberger, M.; Kogler, F. R.; Jupa, M.; Gross, S.; Fric, H.; Kickelbick, G.; Schubert, U. Can the Clusters Zr6O4(OH)4(OOCR)12 and [Zr6O4(OH)4(OOCR)12]2 be converted into each other? *European Journal of Inorganic Chemistry* **2006**, *2006*, 3283–3293.

(S2) Valenzano, L.; Civalleri, B.; Chavan, S.; Bordiga, S.; Nilsen, M. H.; Jakobsen, S.; Lillerud, K. P.; Lamberti, C. Disclosing the complex structure of UiO-66 metal organic framework: a synergic combination of experiment and theory. *Chemistry of Materials* **2011**, *23*, 1700–1718.

(S3) Dai, S.; Simms, C.; Dovgaliuk, I.; Patriarche, G.; Tissot, A.; Parac-Vogt, T. N.; Serre, C. Monodispersed MOF-808 nanocrystals synthesized via a scalable room-temperature approach for efficient heterogeneous peptide bond hydrolysis. *Chemistry of Materials* **2021**, *33*, 7057–7066.
